# Special Issue “Oxidative Stress in Aging and Associated Chronic Diseases”

**DOI:** 10.3390/antiox11040701

**Published:** 2022-04-02

**Authors:** Cristina Mas-Bargues, Matilde Alique, Mª Teresa Barrús-Ortiz, Consuelo Borrás, Raquel Rodrigues-Díez

**Affiliations:** 1 Grupo de Investigación Freshage, Departamento de Fisiología, Facultad de Medicina, Universidad de Valencia, 46010 Valencia, Spain; 2 Instituto Sanitario de Investigación INCLIVA, 46010 Valencia, Spain; 3 Centro de Investigación Biomédica en Red Fragilidad y Envejecimiento Saludable, Instituto de Salud Carlos III (CIBERFES, ISCIII), 28029 Madrid, Spain; 4 Departamento de Biología de Sistemas, Universidad de Alcalá, 28871 Madrid, Spain; 5 Instituto Ramón y Cajal de Investigación Sanitaria (IRYCIS), 28034 Madrid, Spain; 6 Área de Fisiología, Departamento de Ciencias Básicas de la Salud, Facultad de Ciencias de la Salud, Universidad Rey Juan Carlos, Avenida de Atenas s/n, 28922 Madrid, Spain; 7 Departamento de Farmacología, Facultad de Medicina, Universidad Autónoma de Madrid, 28029 Madrid, Spain; 8 Instituto de Investigación Hospital La Paz (IdiPAZ), 28046 Madrid, Spain; 9 Centro de Investigación Biomédica en Red en Enfermedades Cardiovasculares (CIBERCV), 08036 Barcelona, Spain

Aging is a risk factor for several diseases, including cardiovascular disease, type 2 diabetes, hypertension, cancer, osteoarthritis, and Alzheimer; oxidative stress is a key player in the development and progression of aging and age-associated diseases.

The collection of studies in this Special Issue, “Oxidative Stress in Aging and Associated Chronic Diseases,” published in *Antioxidants* (available online: https://www.mdpi.com/journal/antioxidants/special_issues/oxidative_stress_chronic_diseases (accessed on 22 March 2022), serves to highlight the current progress and compiles new contributions that describe novel mechanisms by which oxidative stress, inflammatory factors and extracellular vesicles (EVs) drive the development or progression of chronic diseases, aging or age-associated diseases, as well as new therapeutic strategies to treat or prevent this pathological status. It comprises a series of five research articles, four review articles, and one brief report, which provide the state of art of oxidative stress in the development and progression of aging and chronic disease.

An interesting study showed that hyperphosphatemia, related to vascular aging in old mice (24 months), is associated with vascular damage, including endothelial dysfunction in mesenteric arteries mediated by a decrease in nitric oxide synthase 3 (NOS3) levels and increased inflammation and fibrosis in the aorta [1]. Furthermore, this aging-associated hyperphosphatemia generates an imbalance in the mechanisms of oxidant production and antioxidant capacity in favor of ROS production in the aorta of old mice. Interestingly, old mice fed with a low phosphate diet for three months showed improvement in vascular parameters, suggesting that dietary control of the phosphate intake diet could prevent premature vascular aging and delay aging-associated diseases such as vasculature aging.

Aging is also associated with the development of insulin resistance and other metabolic alterations due to increased visceral adiposity and a decrease in the amount of brown adipose tissue and muscle mass, known as sarcopenia. Nutraceuticals and functional foods (one or more compounds with biochemical and physiological functions beneficial to human health) could increase accessibility to treat these chronic conditions in older people worldwide. In their research paper, González-Hedström et al. [2] describe the beneficial effects of an olive leaf extract (OLE) supplied in a diet for 21 days to ageing rats. OLE treatment increases gastrocnemius weight, decreases the inflammatory status of skeletal muscle and reduces sarcopenia by decreasing myogenin, myostatin, and atrogenes (defined as a gene systematically up- or down-regulated in several catabolic situations) expression and maintaining a better skeletal muscle condition in treated old animals. The research paper showed that the beneficial effects of OLE treatment in adipose tissue and skeletal muscle in old rats are mainly due to its anti-inflammatory effects by increasing anti-inflammatory and decreasing pro-inflammatory markers. Extract administration to aged rats increases the insulin sensitivity in adipose tissue and skeletal muscle and attenuates sarcopenia by reducing the gene expression of pro-inflammatory cytokines and muscle atrophy markers. Thus, it may be a good candidate for the treatment and/or prevention of muscle loss in aged individuals.

The study performed by Marquez-Exposito et al. [3] aimed to unravel the process and molecular mechanisms driving the appearance of chronic kidney disease. To this end, the authors monitored the kidneys of mice from 3 to 18 months old. They found that the process starts at 12 months old, with the appearance of cellular senescence characteristics in nephrotic cells, such as the activation of the DNA damage response. The process was followed by inflammation, mainly driven by the senescence-associated secretory phenotype (SASP) and the appearance of infiltrating inflammatory cells, leading to chronic inflammation. At the same time, oxidative stress was observed through the activation of nuclear receptor factor 2 (NRF2) antioxidant defenses. Moreover, these processes were accompanied by a downregulation of the klotho factor. Finally, oxidative damage to biomolecules, more specifically to lipids, was only visible in mice of 18 months old. These findings suggest that senescence and oxidative stress are early drivers of kidney damage progression and could be used as early biomarkers.

The purpose of Bárcena et al.’s [4] study was to elucidate the molecular mechanisms driving a hepatic loss of function during aging. They reported downregulated antioxidant levels and increased oxidative damage to lipids during aging, thereby suggesting oxidative stress as the original cause. Moreover, they also prove an exacerbated inflammatory response during aging, as measured by interleukins IL-6 and IL-10 and tumor necrosis factor α (TNF-α). Interestingly, they assessed the effect of fasting in these two processes in both young and old mice, and they concluded that prolonged fasting in aged rats weakens the liver’s capacity to respond to refeeding. To support this hypothesis, the authors offer an in-depth analysis of the hepatic nuclear proteome and found out that starvation at advanced ages reduced the expression of antioxidant proteins and increased oxidative damage to biomolecules. Thus, the authors suggest that prolonged fasting is detrimental for older animals. 

These two previous studies focus on the loss of function of two very important organs: the liver and the kidneys. The main idea that can be extracted from these studies is that oxidative stress is the trigger and the driver of the age-related loss of function in both organs, which is followed by inflammation. Therefore, it would be interesting to know whether oxidative stress starts simultaneously in both organs and whether there is any kidney–liver communication (maybe through EVs) involved in the development of the organ dysfunction. 

Not only mammals but also all organisms are affected by aging. In this sense, this Special Issue includes a fascinating study of how aging affects the ability of *Drosophila melanogaster* to tolerate various types of stress factors [5]. In this study, Belyi et al. observed an age-dependent decrease in survival to all stressors employed as the strongest changes for oxidative and genotoxic stressors. Moreover, they observed that young *Drosophila* females have higher stress resistance than males. Based on their results, they proposed an algorithm to calculate the biological age of *Drosophila* by using a two-parameter survival curve model exploring the survival curves for CdCl_2_ and ZnCl_2_ exposition, form males, and paraquat and NaCl survival curves for females. 

Excellent reviews are also included in this Special Issue, emphasizing the importance of oxidative stress in aging and associated chronic diseases. They also highlight the role of other novelty mediators such as EVs as a new therapeutic approach/tools or target to prevent chronic diseases such as cardiovascular diseases, chronic kidney diseases, diabetes, and pathologies related to aging. 

In their work, Amor et al. [6] presented a thorough revision of the contribution of the antioxidant compound ellagic acid (EA) to limiting the diabetes burden. EA is obtained by intestinal hydrolysis of the polyphenol’s family ellagitannins presented in several fruits and seeds, such as pomegranate, walnuts, and berries. In this review, the authors summarized the research conducted on EA, in isolation, extracts, or enriched EA preparations, in Type 2 diabetes experimental models and clinical trials. Among all the effects reported, they highlight the effects of EA on glucose metabolism, inflammation, oxidation and glycation, and diabetic complication, including micro- and macrovascular complications and neurological complications. Furthermore, conclusions obtained from in vitro and in vivo experimental models supported the anti-inflammatory, antioxidant, and anti-glycation role of EA in diabetes. Moreover, in these experimental models, EA supplementation improves the glucose metabolism and diabetic-associated complications, including diabetic nephropathy and retinopathy, atherosclerosis, cardiopathies, and some neurological complications, clearly showing the beneficial effect of this compound in diabetes treatment. However, clinical results are weaker and only confirm the antioxidant and anti-inflammatory properties of EA, while its effects on glycemic control and diabetes-associated complications remain unclear. The authors pointed out that this could be because clinical research is incipient and mainly involves non-randomized and low-powered studies. Therefore, it is necessary to employ well-designed human randomized controlled trials to fill the gap between experimental and clinical research. 

Gonzalez-Moro et al. [7] reviewed the role of the NLRP3 inflammasome in age-associated diseases such as cardiovascular disease, focusing on the mechanisms by which the NLRP3 inflammasome contributes to the genesis and progression of vascular diseases, including vascular inflammation, oxidative stress, and vascular cells senescence, which is the principal hallmark of vascular aging. In addition, the authors summarized current pharmacological therapeutic tools (preclinical, clinical trials, and approved drugs) against the NLRP3 inflammasome. Pointed targeting of the NLRP3 inflammasome by pharmacological interventions could be critical to mitigating the activation of vascular disease. 

In their exciting review [8], Lushchak et al. focus on middle age as a critical point for energy reorganization and redox homeostasis of the brain and its immune system. However, contrary to expectations, changes with aging do not progress linearly with age. Therefore, the authors describe the main pathways leading to brain ROS/RNS production and the most relevant defense systems. They also summarize the recent literature on the changes in the energy/ROS homeostasis and the activation state of the brain’s immune system at midlife. Finally, they also discuss the role of calorie restriction as a readily available and cost-effective lifestyle intervention. 

The review article from the editors of this Special Issue, Mas-Bargues et al. [9], discusses EVs’ role in oxidative stress and inflammation during cardiorenal syndrome development and ageing. The review describes how increased oxidative stress, cell senescence, and chronic inflammation promote cardiovascular and renal diseases as the paradigm of age-related chronic disease. Among these mechanisms, EVs and bioactive molecules secreted by senescent cells produce a pro-inflammatory milieu that promotes disease and ageing. In addition, the review emphasizes the therapeutical effect of molecules that slow down senescence and reset the redox state, and the role of EVs as drug carriers, biomarkers, and drug targets. 

Finally, in this brief report, Martinez de Toda et al. [10] highlight the role of 20S proteasome activity in aging and longevity. The 20S proteasome is involved in the degradation of oxidized proteins. They show two different proteasome activities (CT-like and C-like) in several tissues (heart, lung, liver, kidney, axillary lymph nodes) and cells (peritoneal leukocytes) from adults, old and exceptionally old BALB/c female mice. Their results show different age-related changes depending on the tissue and the activity considered. Interestingly, they find that long-lived mice display maintained proteasome activities, suggesting that the preserved 20S proteasome is associated with successful aging. 

All these Special Issue articles illustrate the deep state of research into oxidative stress in aging and associated chronic diseases. It will provide a broad overview of cutting-edge strategies to counteract the onset of chronic diseases associated with aging and identify several factors involved in developing these pathologies. Moreover, this Special Issue will be helpful to researchers in the development of innovative approaches and research findings into new preventive or therapeutic strategies that may lead to a clinical translation soon. Therefore, we hope that this Special Issue of *Antioxidants* will interest many readers.
